# Shifts in trait-based and taxonomic macrofauna community structure along a 27-year time-series in the south-eastern North Sea

**DOI:** 10.1371/journal.pone.0226410

**Published:** 2019-12-18

**Authors:** Julia Meyer, Ingrid Kröncke

**Affiliations:** 1 Marine Research, Senckenberg am Meer, Wilhelmshaven, Germany; 2 Institute for Chemistry and Biology of the Marine Environment, Benthic Ecology, Oldenburg, Germany; Universidad de Antioquia, COLOMBIA

## Abstract

Current research revealed distinct changes in ecosystem functions, and thus in ecosystem stability and resilience, caused by changes in community structure and diversity loss. Benthic species play an important role in benthic-pelagic coupling, such as through the remineralization of deposited organic material, and changes to benthic community structure and diversity have associated with changes in ecosystem functioning, ecosystem stability and resilience. However, the long-term variability of traits and functions in benthic communities is largely unknown. By using abundance and bioturbation potential of macrofauna samples, taken along a transect from the German Bight towards the Dogger Bank in May 1990 and annually from 1995 to 2017, we analysed the taxonomic and trait-based macrofauna long-term community variability and diversity. Taxonomic and trait-based diversity remained stable over time, while three different regimes were found, characterised by changes in taxonomic and trait-based community structure. Min/max autocorrelation factor analysis revealed the climatic variables sea surface temperature (SST) and North Atlantic Oscillation Index (NAOI), nitrite, and epibenthic abundance as most important environmental drivers for taxonomic and trait-based community changes.

## Introduction

### Diversity and ecosystem function research

While studying ecosystems and their inherent communities, former studies frequently focused on taxonomic descriptions [1–3]. However, not all species are equally important for ecosystem processes and stability [4–6]. Taxonomic structures give only restricted information on *ecosystem functioning*, which are processes accounting for fluctuations of organic matter, nutrients, and energy flows of environments, including primary production, nutrient cycling, and decomposition and on *ecosystem services*, which summarise the benefits people obtain from ecosystems [7, 8]. Consequently, understanding which role species or communities play in an ecosystem and how long-term changes in diversity, and thus in functional traits, can affect ecosystem functioning or at least ecosystem services, seem to be one of the most challenging research items, not only in benthic research [8, 9].

Latest diversity and ecosystem functioning (BEF) research stated that diversity is not a simple product of the physical and chemical parameters of an ecosystem, rather than an important factor, which controls ecosystems [10]. In contrast to the taxonomic identity, trait groups which contribute similarly to ecosystem functioning are directly connected with ecosystem processes [11, 12]. Thus, recent studies increasingly addressed the composition of traits and functions in ecosystems [9, 13, 14]. For the benthic ecosystem, often a few key species drive prevailing processes [15]. A decrease or loss of these species can cause changes in ecosystem function [8]. Otherwise, an increase in species richness can lead on to increasing ecosystem functions, but also to a high level of redundancy in functional traits, resulting in increased ecosystem stability [16]. To examine redundancy of traits and functions, the ratio between the taxonomic and trait-based diversity can be used [17, 18]. There are several studies on functional composition and diversity of temperate shallow-water marine ecosystems [19–21], of pacific fjords [22] and of different deep-sea areas [23] such as hydrothermal vents [24], and the Mediterranean [25]. However, only a few studies connect taxonomic and trait-based structures [17, 26–28].

### Bioturbation and traits of macrofauna species

Macrofauna species are placed at the upper layers of the sediment and at the water-sediment interface, and represent an important element of benthic-pelagic coupling [28]. Macrofauna species act as food resources for larger benthic species, epifauna, or demersal fish species, while they are feed on smaller organisms such as meiofauna and bacteria, defecations from all trophic levels, and from benthic and pelagic phytoplankton [29, 30]. Feeding and foraging activities of macrofaunal species, summarised by the term *bioturbation*, are important for the remineralization of deposited organic material [31–33].

The theoretical community bioturbation potential (BPc) is a proxy for macrofauna-environment interaction, reflected in the biogenic modification of the sediment through particle reworking and (water) movements [34, 35]. Even if BPc is an estimate of the possible potential of a community to bioturbate and not a direct measurement of a defined process, it is the most valuable method for already existing data [36]. Next to biomass and abundance, BPc includes the traits sediment reworking and mobility of benthic organisms, which are most important when describing macrofauna sediment interaction, as a consequence of mobility, feeding mode, or burrowing activities [34, 35, 37].

Several studies have used biological trait analysis (BTA) to analyse functionality and functional diversity of communities[13, 18, 38]. These studies include different traits such as adult longevity, reproductive technique, adult movement, or relative weight [38, 39]. In contrast to aforenamed traits, the traits sediment reworking and mobility are species specific and well known for most of the benthic species of the south-eastern North Sea in contrast to e. g. longevity or reproductive technique.

Furthermore, focus of the present study lay on bioturbation-related traits, because bioturbation is directly linked with ecosystem functioning. Thus, we are assuming that BPc and related trait classification [34, 35] can be used as a functional, trait-based classification for describing most important environmental interactions of macrofauna communities [36, 40].

### Long-term changes in taxonomic and functional macrofauna community variability

Significant long-term changes to taxonomic community variability in marine ecosystems have been documented worldwide [41–43], including in North Sea pelagic [44–46] and benthic [47–49] communities.

Large-scale studies on the taxonomic variability of macrofauna communities in the south-eastern North Sea revealed four nearly-stable taxonomic, abundance-based communities, structured largely by environmental factors sediment composition, depth, and salinity [29, 50, 51]. Between 1986, 2000, and 2010–2015 basic changes in these communities were found, driven by changes in SST and phytoplankton PP [51].

Recent large-scale studies on trait-based benthic communities of the south-eastern North Sea revealed three spatially different communities in 1986 and 2015, with a spatial extend similar to abundance-based communities [36]. Studies of Meyer et al. [36] revealed distinct changes of these three stable trait-based macrofauna communities in the south-eastern North Sea, which were driven by a decrease in food availability due to fluctuations in nitrogen to phosphorus (N:P) ratios [52] and decreasing riverine nutrient input, synchronous to SST changes. However, there is a lack in continuous long-term studies of functional variability, to investigate the functional community variability and diversity, and to assess causes and consequences of changes for the marine and benthic ecosystem. Few studies indicated distinct spatial [53] and long-term variability [14, 54] in taxonomic and trait-based community structures, which were related to anthropogenic and natural factors such as sediment properties, fishing pressure, depth, and temperature [14]. In the Baltic Sea, Törnroos et al. [55] found distinct long-term trends of two key organismal groups, fish and zoobenthos, over a 40 year period. A similar timing of changes in fish and macrofauna were found, amongst others in the early 1990s and the late 2000s [56].

Numerous long-term studies linked changes in the marine ecosystem to temperature and climate parameters such as climate regime shifts, increasing sea surface temperature (SST), cold winters, or the North Atlantic Oscillation Index (NAOI) [e.g. 47, 57–59]. Since 1950, a mean SST increase by 1.5–1.8°C of the southern North Sea was found [60], which was connected with changing patterns of mainly cold-temperate species [3, 48], but also with mass occurrences of opportunistic species [51]. In the Wadden Sea an SST increase by around 1.5°C was found [61], connected with changes of abundance and distribution of demersal fish species [44, 59]; but also, for long-term changes in intertidal taxonomic and trait-based macrofauna community variability [62, 63]. After the cold winter in 1995/96, Reiss et al. [64] found several short-term changes in the benthic communities along a transect of the south-eastern North Sea. Similar changes were found in macrofauna communities of shallower coastal regions [57] and in epibenthic communities of the south-eastern North Sea [65].

Basic changes in the marine environment, resulting in a reorganisation of the community structure are termed as “biological regime shifts” (BRS). These changes are often connected with synergistic and reinforcing effects of climate, temperature, and anthropogenic drivers [43, 58, 66, 67]. Scheffer et al. [68] defined three types of BRS. A smooth BRS is characterised by a linear relationship between driver and response variable. In 2001/02, a climate regime shift led on to an abrupt BRS, which expressed in a non-linear relationship between driver and response variables [57, 69]. In this instance the NAOI [70] was used as a reference factor for identifying responses of the marine environment. And competingly, within a discontinuous BRS there are alternative stable states. These changes are based on taxonomic structures, which provide more insight into structural ecosystem changes than in functional changes. Adding trait-based information into analyses could highlight changes in the functionality of an ecosystem.

By using this almost continuous 27-years benthic time series of four stations along a transect in the south-eastern North Sea, we aim to analyse and compare the long-term variability of taxonomic and trait-based community structure in relation to environmental parameters. We hypothesize 1) that temporal taxonomic and trait-based community structure and diversity are congruent; 2) that changes in the trait-based community structure did not lead to changes in stability and resilience of the bioturbation potential, and 3) that changes in trait-based community structure were not influenced by environmental parameters such as decreasing nutrient supply, and hydroclimatic changes, such as the cold winter in 1995/96 or the climate regime shift in 2000/2001.

## Material and methods

### Study area and sampling

**Fig 1:**
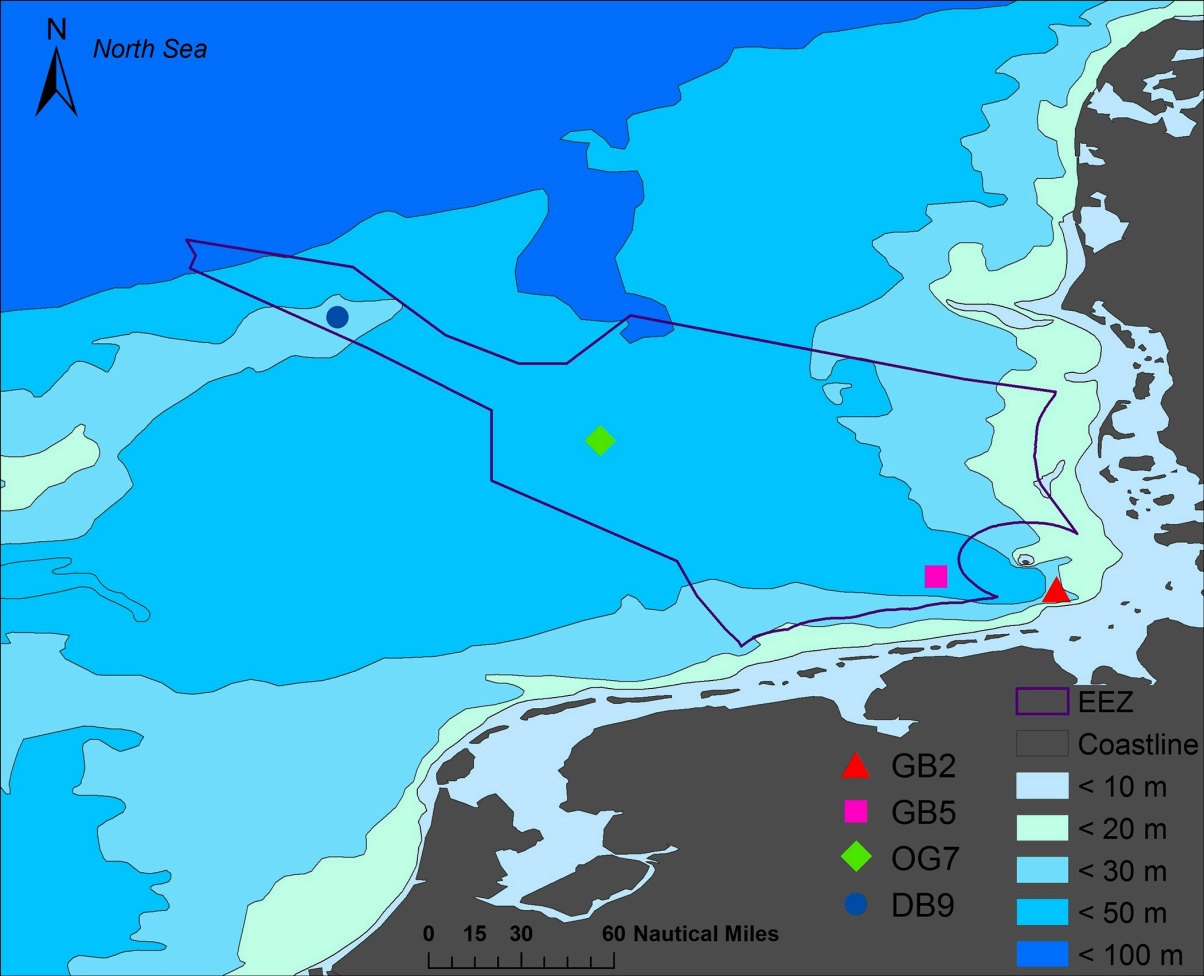
Study area in the south-eastern North Sea below the 50 m depth line. The sampling stations are located in the German Bight (GB2 and GB5), the Oysterground (OG7), and the Dogger Bank (DB9) in the German Exclusive Economic Zone (EEZ).

The present study is part of the Senckenberg Long Term Ecological Research (LTER) Benthos Observatory. The transect involves four stations at the German Exclusive Economic Zone (EEZ), from the inner German Bight (GB2 and GB5), along the Oysterground (OG7) towards the Dogger Bank (DB9) (Fig 1), which were sampled in 1990 and from 1995 to 2017. Excepting the shallower Dogger Bank with 30 m at DB9, depth increases with distance from the coast, between 27 m at GB2 to 40 m at OG7 (Fig 1).

At each station 3–5 replicates were taken annually in May from 1996 to 2017 with a 0.1 m^2^ Van Veen grab, except in March 1990 (0.0122 m^2^ box corer) and in May 1995 (0.2 m^2^ Van Veen grab for stations OG7 and DB9) [64]. Samples were sieved over 1 mm mesh size and fixed in 4% buffered formaldehyde. Taxa were determined up to species level, counted, and weighed. Missing biomass data were added by a biomass index for the south-eastern North Sea [51].

### Environmental parameters

**Fig 2:**
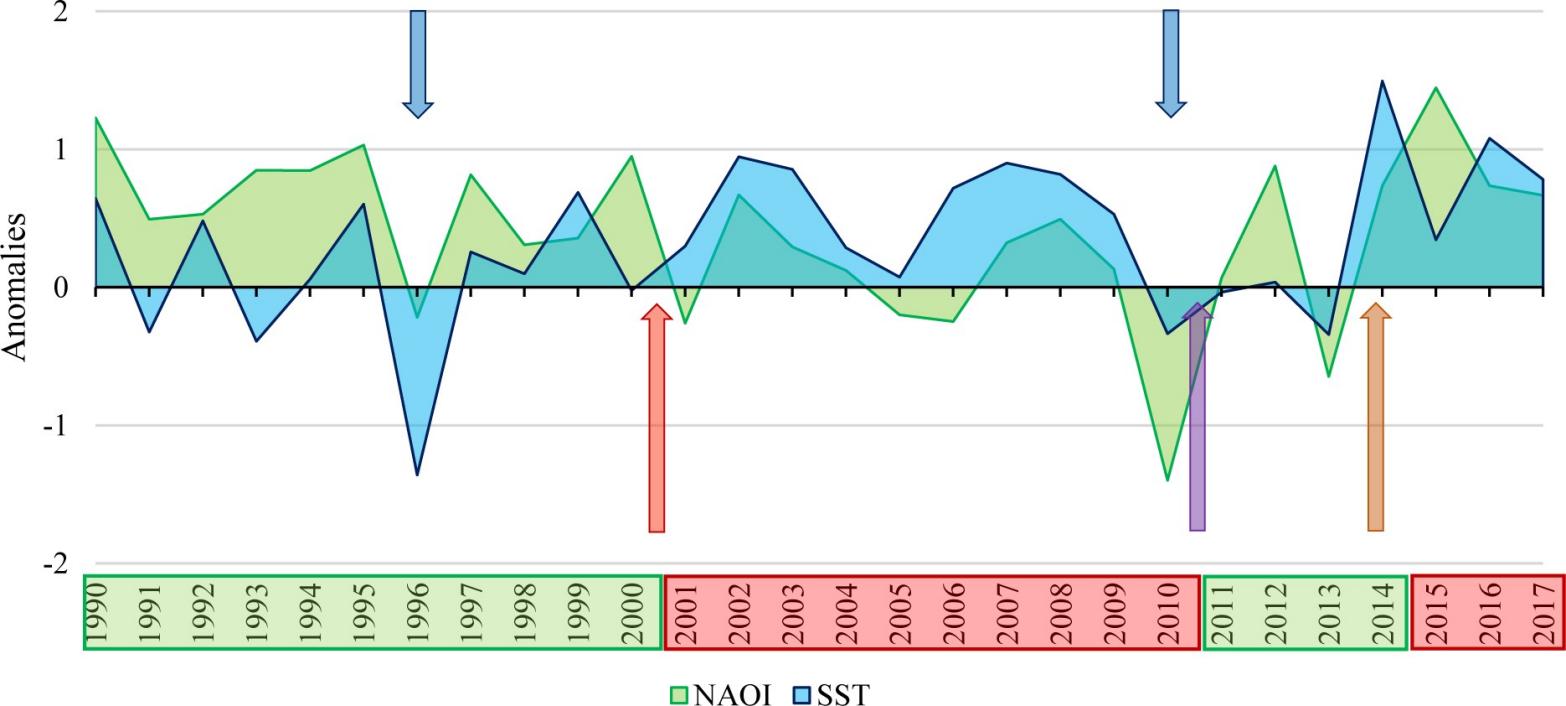
Annually sea surface temperature (SST) and North Atlantic Oscillation Index (NAOI) anomalies from 1990 to 2017. SST of one station (WB: White Bank, located at OG7) is shown exemplarily for the study area in the south-eastern North Sea. Boxes–green: highly variable phase, red: predominantly positive anomalies; arrows–red: abrupt biological regime shift, purple: biological regime shift, orange: hydroclimatic shift, blue: cold winters.

Weekly sea surface temperature (SST in°C) data of the four stations were provided by the Federal Maritime and Hydrographic Agency of Germany (BSH/ https://bsh.de). Yearly and winter North Atlantic Oscillation Index (NAOI) were taken from https://climatedataguide.ucar.edu [71]. Long-term variability of annually SST and NAOI anomalies are shown in Fig 2.

Nutrient loads (phosphate PO_4_; in mg P/L of river surface waters after filtration and nitrite NO_2_; in mg N/L of river surface after filtration) were used as a proxy for phytoplankton primary production (PP), because of the significant correlation between nutrient intake and PP in the south-eastern NS [72]. Nitrite data from river Rhine, measured at station Lobith, Netherlands were used in the present study (extracted from the Dutch ministry of Infrastructure and the Environment, Rijkswaterstaat; https://waterinfo.rws.nl).

For sediment analysis a separate sediment grab was taken at each sampling and station. Shell and coarse sand was sampled off each sample and weight, giving the shell content. Then samples were sieved over mesh sized of 63 μm to determine mud and sand content.

### Epibenthic data

Abundance data of characteristic and dominant epibenthic species from the south-eastern North Sea were used as a proxy for feeding pressure of higher trophic levels [65, 73, 74]. Only epibenthic species, nourishing mainly from macrofauna were extracted for this analysis. Samples were taken with a standardised 2 m beam trawl, fitted with a 20 mm net and a cod end of 4 mm mesh size [65, 74]. A priori autocorrelation analysis of epibenthic mean abundance data and other environmental parameters was processed, to exclude a high autocorrelation between the parameters.

### Community bioturbation potential and trait groups

The BPc was determined according to Solan et al. [34] and Queirós et al. [35].

$${BP}_{C}=\sum_{i=1}^{n}\sqrt{\frac{Bi}{Ai}}\;x\;Ai\;x\;Mi\;x\;Ri.$$

Macrofauna biomass (Bi) and abundance (Ai) of taxon i were used. Each taxon i was classified into categorical scales of Mi (mobility) and Ri (sediment reworking) (Table 1). Combining Mi and Ri, trait groups were formed (e.g. B/SM biodiffusors with slow free movement through the sediment matrix).

**Table 1:** Abbreviations (A) and scores for mobility (Mi) and sediment reworking (Ri) traits for benthic taxa in the southern North Sea according to Queirós et al. [35].

Score	Mi	A	Ri	A
**1**	living in a fixed tube	FT	-	
**2**	limited movement	LM	surficial modifiers	S
**3**	slow free movement through the sediment matrix	SM	upward/downward conveyors	U
**4**	free, three-dimensional movement	FM	biodiffusors	B
**5**	-		regenerators	R

### Shannon Diversity Index and FD/SD ratio

Taxonomic (SD)- and trait-based (FD) Shannon Diversity Index H’ log_(e)_ per 1 m^2^ were determined. The Shannon Diversity Index uses the total number of taxa/trait groups *X* and the proportion of the total abundance/BPc of each taxa/trait group *t* (P_i_) $H^{\prime }=-\sum_{t=1}^{x}P_{t}*\log_{e}P_{t}$ [75].

The FD/SD ratio can be used as a measurement for trait redundancy, where a higher FD/SD ratio indicates a lower trait redundancy and vice versa [17, 18].

#### Chronological clustering

A chronological clustering was performed, based on mean abundance per taxa and year, based on mean BPc per trait group and year, and based on mean SST and NAOI per year. Chronological clustering is designed for gradual clustering of time series. A connectedness level of 0.5 and a fusion level alpha of 0.1 were used. Small alpha levels, such as used in the present analysis provide a bird’s eye overview, visualizing the most important breaks in time series, while larger alpha values (0.2 up to 0.9) provide more detailed information [76, 77].

### Abundance- and trait-based long-term analysis

For each station annually mean abundance, mean biomass, mean BPc, abundance (SD)- and trait-based (FD) Shannon Diversity Index H’, and FD/SD ratio are given per m^2^, taxa numbers are given per 0.1 m^2^.

#### Min/max autocorrelation factor analysis (MAFA)

An abundance- and trait-based MAFA was accomplished for each station, using the software package Brodgar (http://ww.brodgar.com). For abundance-based analysis characteristic species of each station were used, for trait-based analysis all trait groups of a station were used. Characteristic species were determined with similarity percentage (SIMPER) analysis, using PRIMER 7 [75]. For each station 20 characteristic species were selected. High correlated (Pearson correlation coefficient > 0.75) species/trait groups were excluded from analysis. MAFA is a type of principal component analysis (PCA) for time series. The MAFA-axis represents the autocorrelation of a variable within a time lag k (k = 1, 2, …). Trends in data are related to highest autocorrelation within time lag 1. The 1^st^ MAFA-axis presents the most common pattern of most variables in the time series. The 2^nd^ MAFA-axis reflects the second most important trend in time series.

#### Canonical correlation analysis

Canonical correlations were used to identify significant relationships between MAFA axis and response variables (abundance/bioturbation data), and further between trends and explanatory variables (environmental data). Abundance, bioturbation, and environmental data were standardized to zero mean. Standard deviation was used for an easier interpretation of the estimated regression parameters.

#### Matrix display analysis

For each station a taxonomic and trait-based matrix display analysis was proceeded with Primer 7, to visualise long-term changes in characteristic species or trait groups by using a shade plot. At each station, characteristic species or trait groups were ordered using hierarchical clustering based on Whittaker's index. It describes similarities between every pair of species or trait group, that have similar patterns of abundance or BPc over samples.

## Results

### Chronological clustering

Using chronological clustering abundance-based analyses revealed three clusters, trait-based analyses revealed six clusters, and SST/NAOI analyses revealed four clusters (Table 2). A shift from the first to the second cluster was found for all three analyses between 1999 and 2001. Within the trait-based analysis two more shifts in 2003 and 2007 were found. In 2010, however, chronological clustering revealed an overall shift from one cluster to another. Altogether, the two simultaneous shifts in around 2000 and in 2010 revealed by the chronological clustering were used as basis for further long-term analyses (Table 2).

**Table 2:** Results of chronological clustering analysis. An alpha level of 0.1 was used. Starting points of clusters are numbered from 1 to 6. Results are shown for abundance-based (AB), Trait-based (TB) and sea surface temperature/ North Atlantic Oscillation Index (SST/NAOI) clustering. Red: Simultaneous changes in AB/TB and SST/NAO which are congruent with the abrupt biological regime shift; purple: Simultaneous changes in AB/TB and SST/NAO which are congruent with the biological regime shift.

	AB	TB	SST/NAOI
1990	1	1	1
1995			
1996			
1997			
1998			
1999	**2**		
2000		**2**	
2001			**2**
2002			
2003		**3**	
2004			
2005			
2006			
2007		**4**	
2008			
2009			
2010	**3**	**5**	**3**
2011			
2012			
2013			
2014			**4**
2015			
2016		**6**	
2017			

### Long-term variability of SST and NAOI anomalies

Since 1990, four different hydroclimatic regimes were found using chronological clustering based on mean winter SST and NAOI, reflected in different SST and NAOI anomalies (Fig 2).

The first regime, between 1990 and approximately 2000/01 was characterized by a high amplitude and variable SST and NOAI anomalies, negatively pronounced in winter and positively in summer, resulting in a warmer phase with mild winters. The second regime, between 2001/02 and 2010/11, was characterised by mainly positive SST and NAOI anomalies in summer and winter, resulted in a warm winter period with an increased storm frequency. The third regime, which started in 2010/11, was characterised by a high amplitude and highly variable SST and NOAI anomalies, comparable to the first regime. The fourth hydroclimatic regime, starting in 2014 was characterized by highly positive SST and NAOI anomalies, comparable with the second regime (Fig 2). In 1995/96 a cold winter was found, characterised by highly negative SST and NAOI anomalies, while the cold winter in 2009/10 was characterised by highly negative NAOI and SST anomalies (Fig 2).

### Taxonomic versus trait-based long-term variability of benthic communities

**Fig 3:**
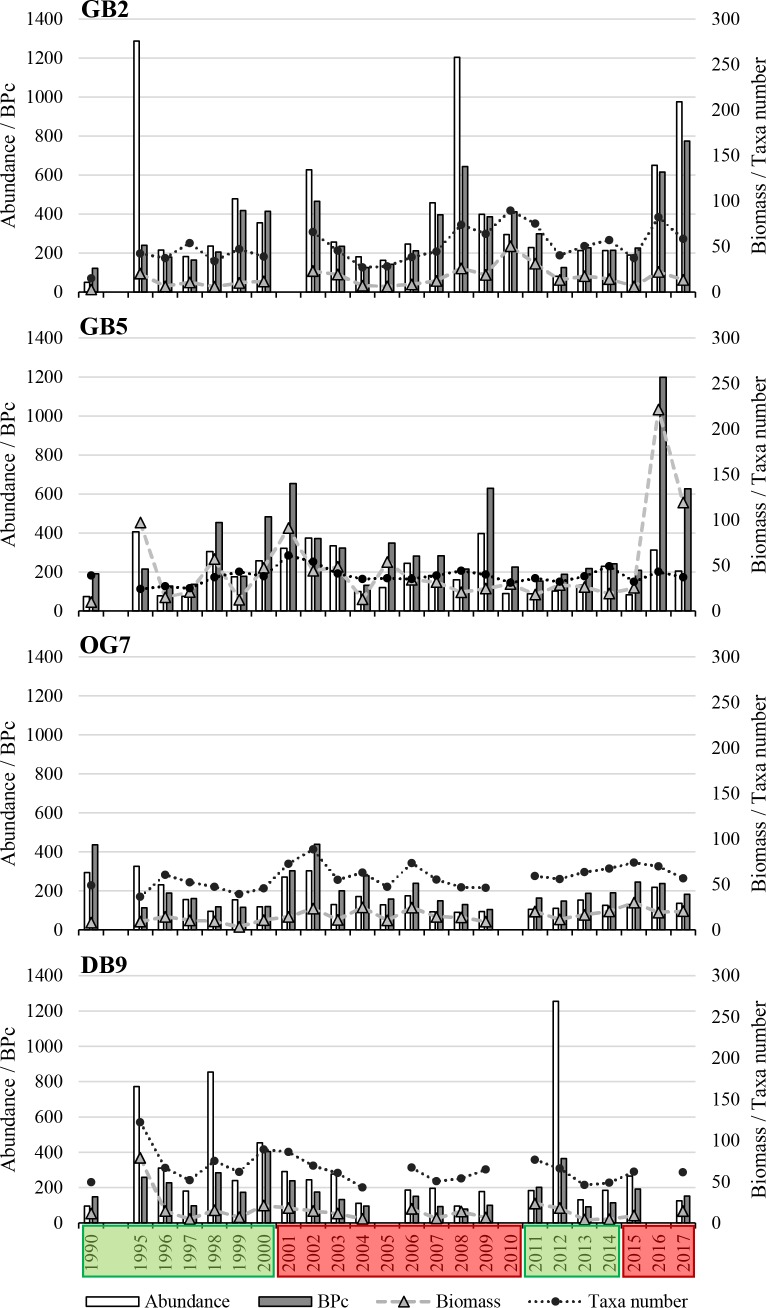
Mean abundance, biomass, and BPc per 1 m^2^ and total taxa number per 0.1 m^2^ per station from 1990 to 2017. Hydrodynamic regimes are shown in boxes–green: highly variable phase, red: predominantly positive anomalies.

**Fig 4:**
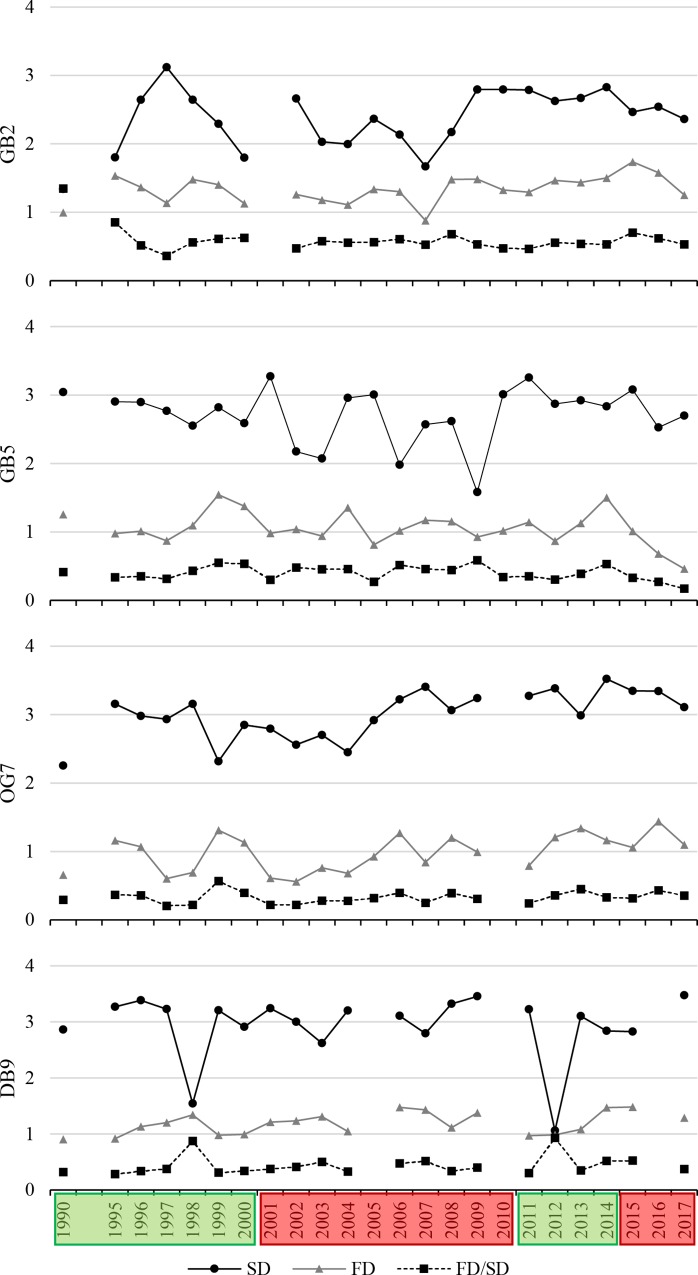
Long-term changes in taxonomic and trait-based diversity. Shown are taxonomic, abundance-based (SD)- and trait-based (FD) Shannon Diversity Index H’ log (e) and FD/SD ratio, of the stations GB2, GB5, OG7, and DB9 from 1990 to 2017. Hydrodynamic regimes are shown in boxes–green: highly variable phase, red: predominantly positive anomalies.

Next to evident gradual spatial differences in abundance, biomass, taxa number, and BPc between the four stations, distinct differences in long-term variability are shown in the present results. Highest interannual variability of all parameters was found at the shallower stations GB2 and DB9, while lowest interannual variability was found at the deepest station OG7 (Fig 3). The taxonomic (SD) and trait-based (FD) diversity and the FD/SD ratio of all stations showed a high annual variability since 1990, but no clear trends or regimes (Fig 4). Generally, three different regimes, adapted from the long-term variability of SST and NAOI are recognizable in the taxonomic and trait-based long-term variability of benthic communities.

**Fig 5:**
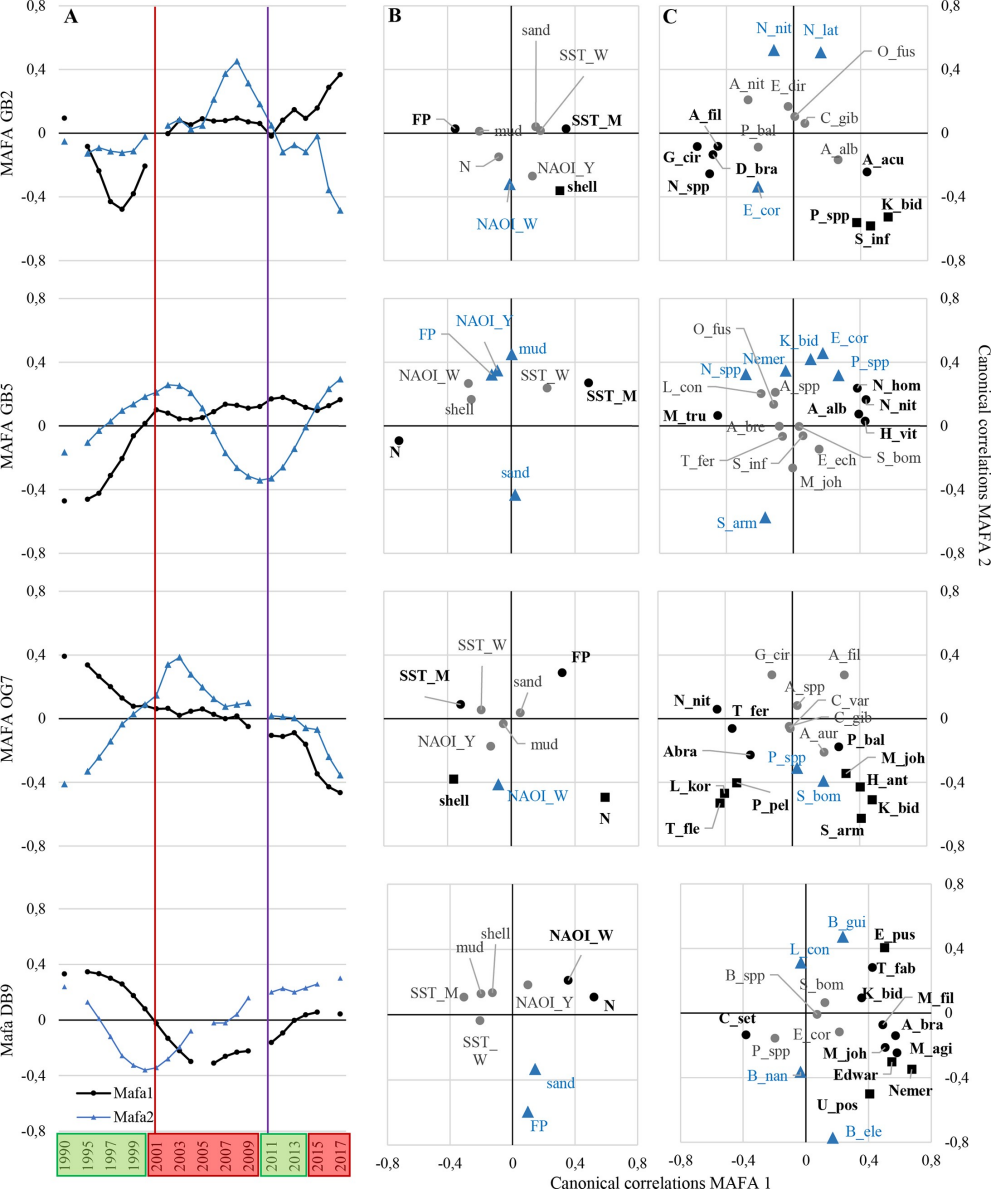
Taxonomic (abundance-based) min/max autocorrelation factor analysis (MAFA) and canonical correlation analysis. (A) MAFA of the stations GB2, GB5, OG7, and DB9 from 1990 to 2017 (1^st^ (black dots) and 2^nd^ (blue triangles) MAFA-axis). Canonical correlations between MAFA-axes and (B) environmental parameters* and (C) characteristic species*. *Black dots indicating a significant correlation with 1^st^ MAFA-axis, blue triangles with 2^nd^ MAFA-axis, black squares with both axes, and grey dots no significant correlation. Red line: Shift between 1^st^ and 2^nd^ regime; Purple line; Shift between 2^nd^ and 3^rd^ regime. Hydrodynamic regimes are shown in boxes–green: highly variable phase, red: predominantly positive anomalies. Abbreviations: (B) *shell*, *mud*, and *sand* content; SST_M/W *annual/winter mean sea surface temperature*; N *nitrite*; NAOI_W/Y *mean winter/annual North Atlantic Oscillation Index*; FP *feeding pressure*—*epibenthic abundance 1*^*st*^ *quarter*, (C) *Abra Abra* spp.; A_acu *Aphrodita aculeata*; A_alb *Abra Alba*; A_aur *Amphictene auricroma*; A_bra *Acrocnida brachiata*; A_bre *Ampelisca brevicornis*; A_fil *Amphiura filiformis*; A_nit *Abra nitida*; A_spp *Amphiura* spp.; B_ele *Bathyporeia elegans*; B_gui *Bathyporeia guillamsoniana*; B_nan *Bathyporeia nana*; B_spp *Bathyporeia* spp.; C_gib *Corbula gibba*; C_set *Chaetozone setosa*; C_var *Chaetopterus variopedatus*; D_bra *Diastylis bradyi*; E_cor *Echinocardium cordatum*; E_dir *Ensis directus*; E_ech *Echiurus echiurus*; E_pus *Echinocyamus pusillus*; Edwar *Edwardsia* spp.; G_cir *Gattyana cirrhosa*; H_ant *Harpinia antennaria*; H_vit *Hyala vitrea*; K_bid *Kurtiella bidentata*; L_con *Lanice conchilega*; L_kor *Lagis koreni*; M_agi *Megaluropus agilis*; M_fus *Magelona filiformis*; M_joh *Magelona johnstoni*; M_tru *Mya truncata*; N_hom *Nephtys hombergii*; N_lat *Notomastus latericeus*; N_nit *Nucula nitidosa*; N_spp *Nephtys* spp.; Nemer *Nemertea* spp.; O_fus *Owenia fusiformis*; P_bal *Pholoe balthica*; P_pel *Phaxas pellucidus*; P_spp *Phoronis* spp.; S_arm *Scoloplos armiger*; S_bom *Spiophanes bombyx*; S_inf *Scalibregma inflatum*; T_fab *Tellina fabula*; T_fer *Tellimya ferrunginosa*; T_fle *Thyasira flexuosa*; U_pos *Urothoe poseidonis*).

**Fig 6:**
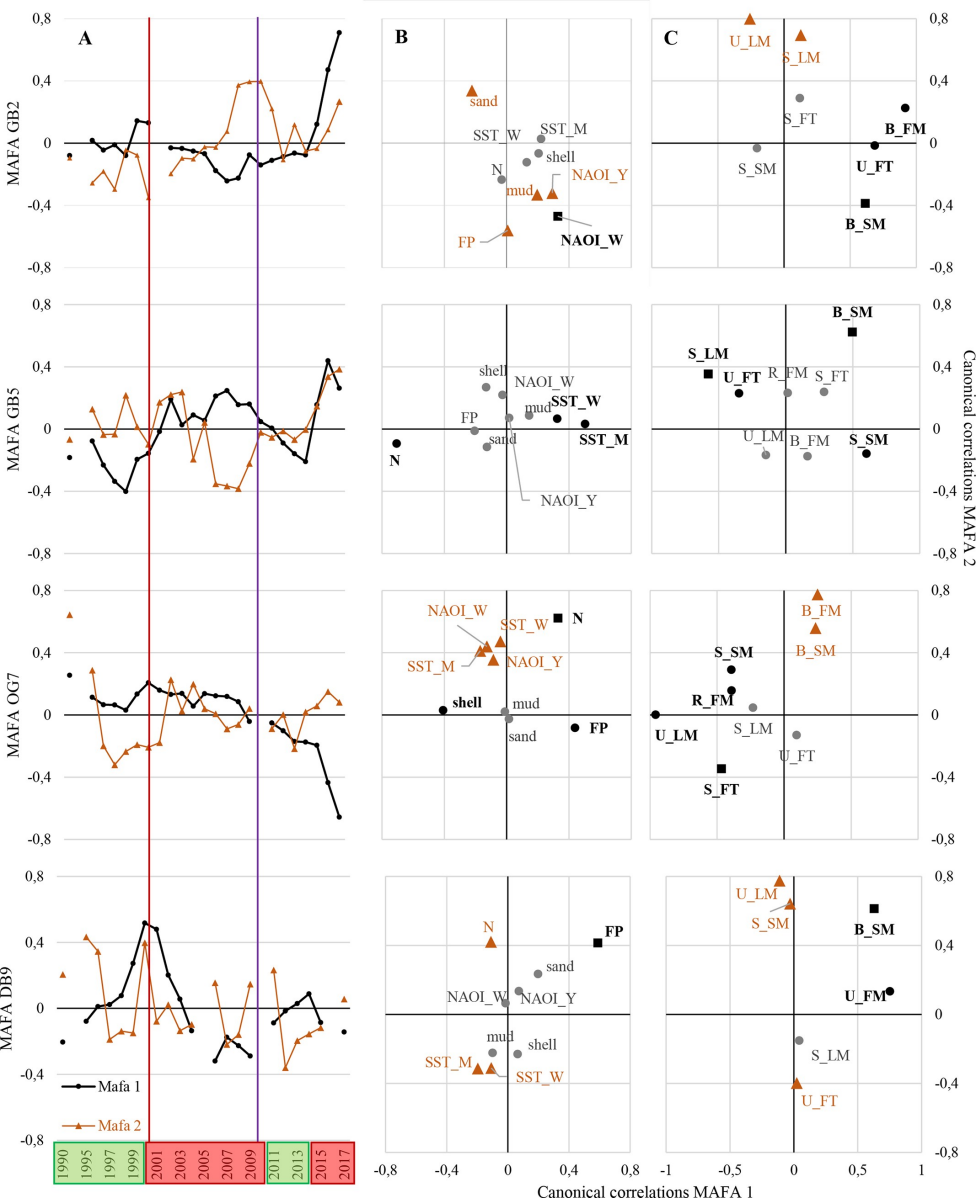
Trait-based min/max autocorrelation factor analysis (MAFA) and canonical correlation analysis. (A) MAFA of the stations GB2, GB5, OG7, and DB9 from 1990 to 2017 (1^st^ (black dots) and 2^nd^ (orange triangles) MAFA-axis). Canonical correlations between MAFA-axes and (B) environmental parameters* and (C) and trait groups*. *Black dots indicating a significant correlation with 1^st^ MAFA-axis, orange triangles with 2^nd^ MAFA-axis, black squares with both axes, and grey dots no significant correlation. Hydrodynamic regimes are shown in boxes–green: highly variable phase, red: predominantly positive anomalies. Red line: Shift between 1^st^ and 2^nd^ regime; Purple line; Shift between 2^nd^ and 3^rd^ regime. Abbreviations: (B) *shell*, *mud*, and *sand* content; SST_M/W *annual/winter mean sea surface temperature*; N *nitrite*; NAOI_W/Y *mean winter/annual North Atlantic Oscillation Index*; FP *feeding pressure*—*epibenthic abundance 1*^*st*^ *quarter*, (C) U_LM upward/downward conveyors / limited movement; U_FT upward/downward conveyors / living in a fixed tube; U_FM upward/downward conveyors / free, three-dimensional movement; S_SM surficial modifiers / slow free movement through the sediment matrix; S_LM surficial modifiers / limited movement; S_FT surficial modifiers / living in a fixed tube; R_FM regenerators / free, three-dimensional movement; B_SM biodiffursors / slow free movement through the sediment matrix; B_FM biodiffursors / free, three-dimensional movement.

A taxonomic and a trait-based MAFA was proceeded separately for each station, revealing several congruent underlying patterns since 1990 (Fig 5 and Fig 6). The trait-based MAFA, especially the 2^nd^ axis, revealed a high interannual heterogeneity, which is not described in the following, because the overall trends followed the trends of the 1^st^ axes.

#### First regime

Between 1990 and 2000/01 highly variable values of abundance, biomass, taxa number, and BPc were found. After the overall decrease in abundance after the cold winter in 1995/96, between 2000 and 2002 an increase was found at stations GB2, GB5, and OG7. At station OG7, the increase resulted in abundances and BPc in 2001, which were twice as high as in 2000 (Fig 3). Considering the taxonomic MAFA, values 1^st^ and 2^nd^ axes of the nearshore station GB2 decreased until 1997, followed by a rapid increase until 2001, while values of station GB5 increased from 1990 until 2000/01, while values of the 1^st^ MAFA axes of the more offshore stations decreased (Fig 5). In the trait-based MAFA, values of the 1^st^ MAFA of stations GB5 and DB9 increased, while values of the 1^st^ MAFA of stations GB2 and OG7 remained stable (Fig 6).

#### Second regime

From 2001/02 onwards, values of abundance, biomass, taxa number, and BPc remained stable over time (Fig 3). In the taxonomic MAFA, values of the 1^st^ axis of stations GB2, GB5, and OG7 remained nearly stable between 2000/01 and 2010/11, while values of DB9 decreased until 2004, followed by a slight increase until 2009 (Fig 5). In the trait-based MAFA, values of GB2 and OG7 decreased slightly, values of DB9 decreased greatly, while values of GB5 remained nearly stable (Fig 6).

#### Third regime

Between 2008 and 2012 an increase in abundance, biomass, taxa number, and BPc similar to 2000 and 2002 was found for stations GB2, GB5, and DB9. It was followed by a stable phase until 2016/17, when abundance, biomass, and BPc of stations GB2 and GB5 increased (Fig 3). Within the third regime values of the taxonomic 1^st^ MAFA axis increased at stations GB2 and DB9, values of the 1^st^ MAFA axis at station GB5 remained stable, while values at OG7 decreased (Fig 5). Similar trends were found for the trait-based MAFA, except for station GB5, where values first deceased until 2014 and subsequently increased (Fig 6).

### Environmental drivers and response variables

**Fig 7:**
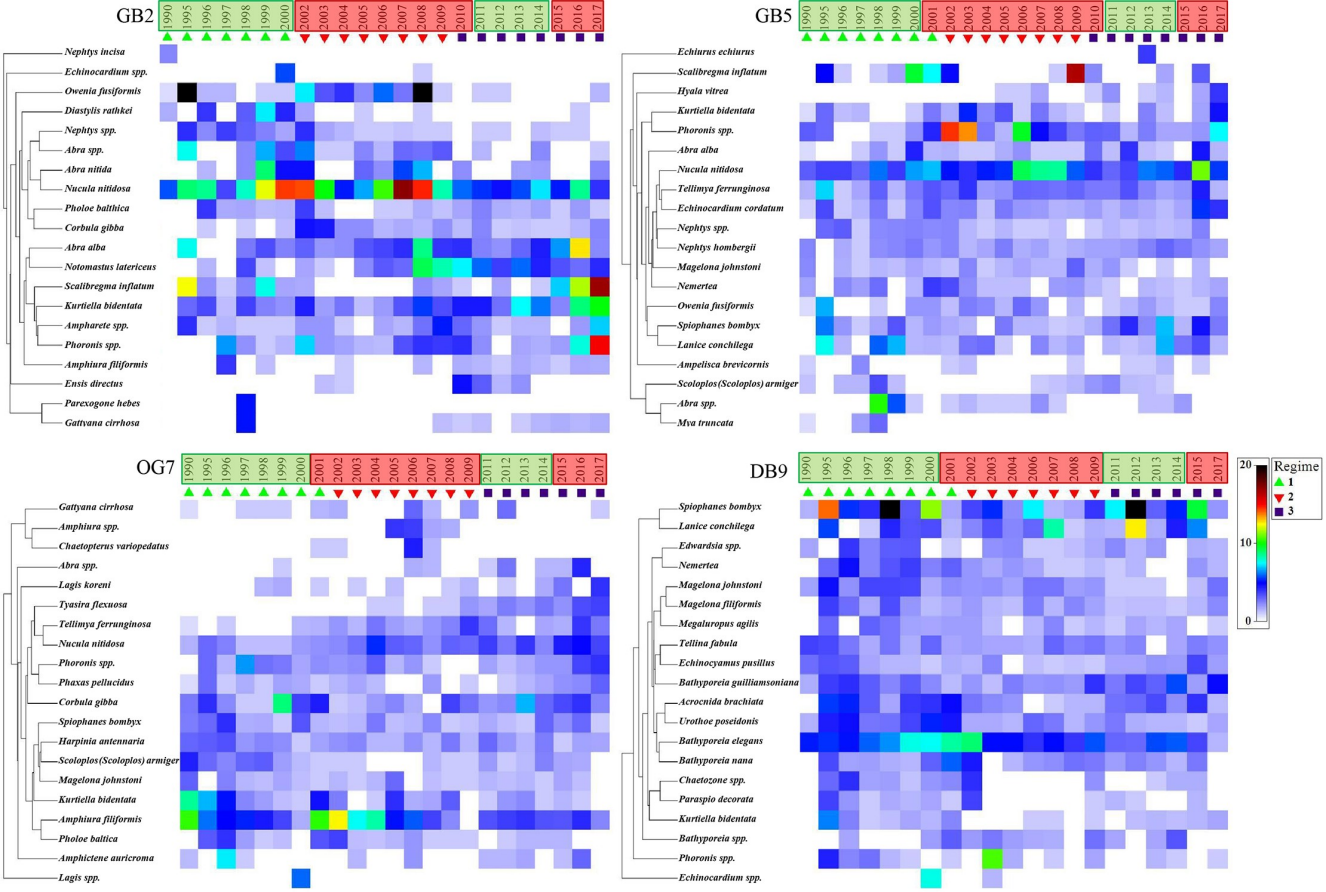
Shade-plot of taxonomic (abundance-based) macrofauna community structure per station. Samples are shown by year, clustered in three regimes, variable sorting was based on a numeric standardised dataset, coloured from 0 (white) to 20 (black). Hydrodynamic regimes are shown in boxes–green: highly variable phase, red: predominantly positive anomalies.

**Fig 8:**
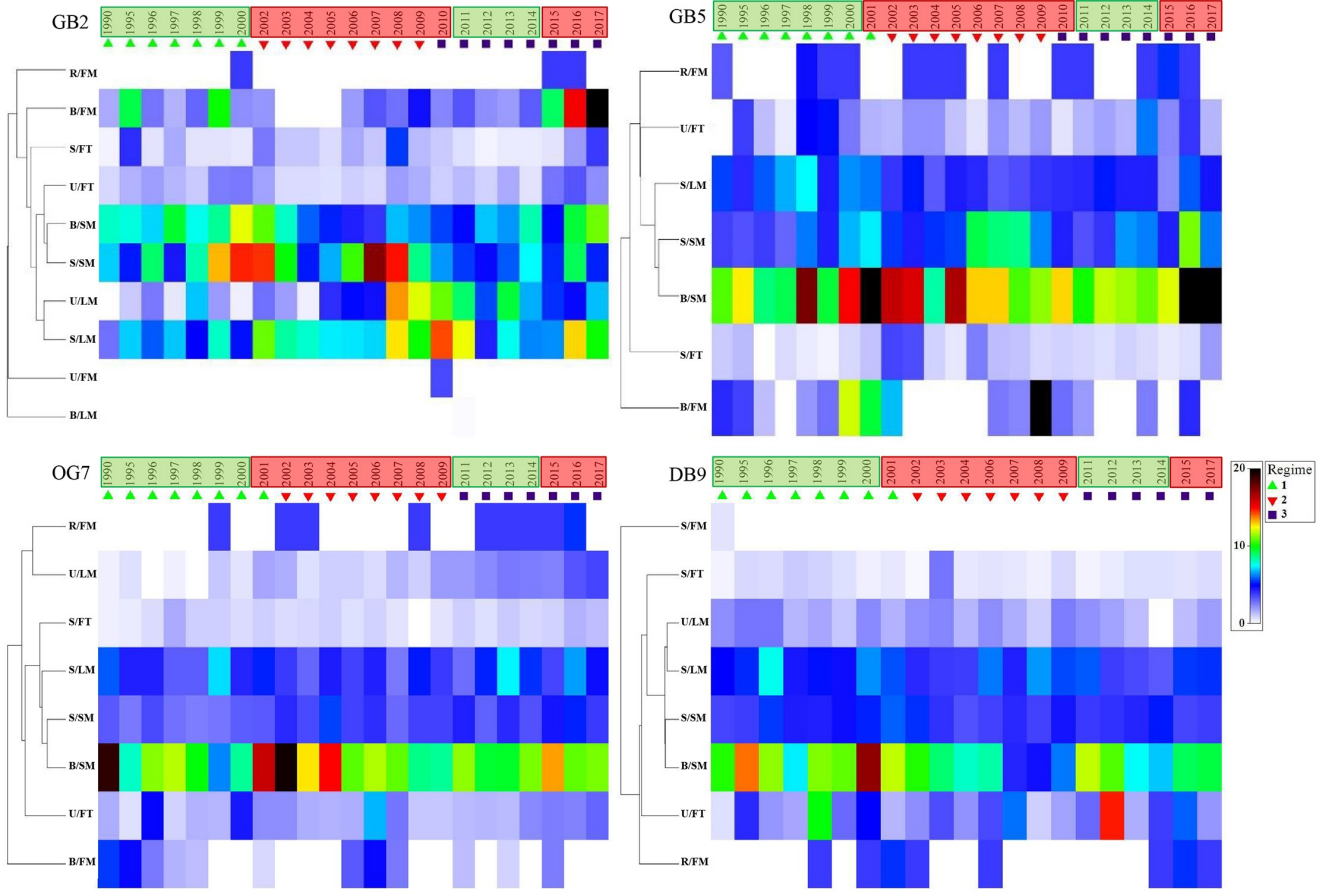
Shade-plot of trait-based macrofauna community structure per station. Samples are shown by year, clustered in three regimes, variable sorting was based on a numeric standardized dataset, colored from 0 (white) to 20 (black). Hydrodynamic regimes are shown in boxes–green: highly variable phase, red: predominantly positive anomalies.

In total 9 environmental driver variables and 20 characteristic species or 8 trait groups as response variables were used for the MAFA. The correlations of driver and response variables were analysed by a canonical correlation analysis (Fig 5 and Fig 6), only the significant correlations with a correlation coefficient > 0.4 are described in the following. The long-term variability of response variables is visualised by using shade plots (Fig 7 and Fig 8). Significantly correlated environmental drivers and response variables differed between the stations and between the taxonomic and trait approaches (Fig 5 and Fig 6).

#### Taxonomic approach

At **station GB2**, the1^st^ MAFA axis was negatively correlated with feeding pressure, positively with shell content. The 2^nd^ MAFA axis was negatively correlated with winter NAOI and shell content (Fig 5). Opportunistic and environmental tolerant species such as *Scalibregma inflatum* and *Phoronis* spp. were positively correlated with the 1^st^ MAFA axis and negatively with the 2^nd^ MAFA axis of GB2. Abundances of these species increased slightly from 1990 until 2017 and were found in highest abundances in 2015–2017 (Fig 7). An increase in abundances of species such as *Gattyana cirrhosa* and *Amphiura filiformis* was found. Both species are sensitive to feeding pressure and food intake were negatively correlate with the 1^st^ MAFA axis. For mud related species such as *Nucula nitidosa* stable abundances and a positive correlation with the 2^nd^ MAFA axis were found (Fig 5 and Fig 7).

At **station GB5** the 1^st^ MAFA axis was positively correlated with mean SST, negatively with nitrite, the 2^nd^ MAFA axis was positively correlated with feeding pressure, mud, and mean yearly NAOI, negatively with sand content (Fig 5). The polychaete *Nephtys hombergii*, the bivalves *Nucula nitidosa* and *Abra alba*, and the gastropod *Hyala vitrea* were highly positive correlated with the 1^st^ MAFA axis of station GB5. All species were found with slight increasing abundances from 1990 until 2017. The bivalve *Mya truncata* was negatively correlated with the 1^st^ MAFA axis. It was only found from 1990 to 1998. The polychaete *Scoloplos armiger* was negatively correlated with the 2^nd^ MAFA axis, it was found in higher abundances from 1990 until 2000 and between 2008 and 2015 (Fig 5 and Fig 7).

The 1^st^ MAFA axis of **station OG7** was positively correlated with feeding pressure and nitrite, negatively with mean SST and shell content. The 2^nd^ MAFA axis was negatively correlated with winter NAOI and in contrast to the 1^st^ axis negatively with nitrite and shell content (Fig 5). Station OG7 was characterised by positive correlations of the 1^st^ MAFA axis and SST tolerant species such as *Magelona johnstoni*, *Harpinia antennaria*, and *Scoloplos armiger*, showing slight decreasing abundances from 1990 until 2017. A negative correlation was found for *Phaxas pellucidus*, *Lagis koreni*, and *Thyrasira flexuosa*. The 2^nd^ MAFA axis was significant correlated with the tube-living *Phoronis* spp. and *Spiophanes bombyx*, for which increasing abundances were found from to 2017 (Fig 5 and Fig 7).

The 1^st^ MAFA axis of **station DB9** was positively correlated with nitrite and winter NAOI. The 2^nd^ MAFA axis was negatively correlated with feeding pressure and sand content. No significant positive correlations were found (Fig 5). At DB9, only 4 taxa were not significantly correlated with the 1^st^ or 2^nd^ MAFA axis. Highest correlation was found for the less mobile *Echinocyamus pusillus and Edwardsia* spp. and the sand-licking amphipod *Urothoe poseidonis*. These species were found in highly variable abundances. The 2^nd^ MAFA axis at station DB9 was highly correlated with the small sand-licking amphipods *Bathyporeia guilliamsoniana*, *B*. *nana*, and *B*. *elegans*, which were found with highest abundances from 1990 to 2002/03 and the tube-living *Lanice conchilega* (Fig 5 and Fig 7).

#### Trait-based approach

The 1^st^ MAFA axis of **station GB2** was positively correlated with winter NAOI. The 2^nd^ MAFA axis was positively correlated with sand content, negatively with feeding pressure, mud content, and yearly NAOI (Fig 6). Highest positive correlation with 1^st^ MAFA of station GB2 was found for trait groups B/FM and B/SM, showing increasing BPc, while the second axis was positively correlated with trait group U/LM, which was found with a high BPc around 2008 to 2010 (Fig 6 and Fig 8).

The 1^st^ MAFA axis of **station GB5** was positively correlated with winter SST and mean annual SST and negatively correlated with nitrite. For the 2^nd^ MAFA axis no significant correlations were found (Fig 6). Highest positive correlation with the 1^st^ MAFA axis of station GB5 was found for trait groups B/SM and S/SM, for both groups high BPc was found around 2000/01 and in 2016/17, trait group S/LM was negatively correlated, found with slight decreasing BPc from 1990 to 2017 (Fig 6 and Fig 8).

The 1^st^ MAFA axis of **station OG7** was positively correlated with feeding pressure and nitrite, negatively with shell content. The 2^nd^ MAFA axis was positively correlated with nitrite, winter and mean SST, and mean annual and winter NAOI (Fig 6). At station OG7 the 1^st^ MAFA axis was negatively correlated with trait group U/LM, for which increasing BPc was found, while the 2^nd^ MAFA axis was positively correlated with trait group B_FM, for which decreasing BPc was found (Fig 6 and Fig 8).

At **station DB9** the 1^st^ MAFA axis was positively correlated with feeding pressure. The 2^nd^ MAFA axis was positively correlated with nitrite and feeding pressure, negatively with mean and winter SST (Fig 6). Highest correlation with the 1^st^ and 2^nd^ MAFA axis of station DB9 was found for trait group B/SM, for which highest BPc was found from 1990 until 2000/01 (Fig 6 and Fig 8).

## Discussion

During the analysis of macrofauna long-term changes along a transect from the German Bight towards the Dogger Bank in May 1990 and annually from 1995 to 2017, congruent changes in taxonomic and trait-based community variability and diversity were found. Overall, taxonomic and trait diversity remained stable over time. Two basic shifts in community structure around 2000 and in 2010 were found, which were concurrent with changes in the hydroclimatic regime of the south-eastern North Sea. However, during a fourth hydroclimatic shift in 2014 we no simultaneous changes in macrofauna community structure. Most important environmental drivers for the taxonomic and trait-based community changes were the environmental parameters sea surface temperature (SST) and North Atlantic Oscillation Index (NAOI), nitrite, and epibenthic abundance.

### Long-term changes in taxonomic and trait-based diversity

The meaning of diversity has changed from a simple product of the physical and chemical parameters of an ecosystem towards an important ecosystem controlling factor [10]. In this context, major importance was attributed to functional diversity, because functional groups which contribute similarly to ecosystem functioning are directly connected with ecosystem processes [11, 12].

Several studies revealed distinct changes in diversity [57, 78] [8, 79]. Results of the present study revealed a lower trait-based than taxonomic diversity, because most benthic species were grouped to a low number of similar functions in contrast to a high number of taxonomic identity [80].

Since 1990, we found no significant long-term changes, neither of taxonomic and trait-based diversity, nor of trait redundancy. This could be hint for the stability of south-eastern North Sea benthic communities, either due to the basic adaption on high disturbance frequency and ecosystem changes or due to resilience and the ability of a fast recovery after disturbance. A stable community is characterised by a high degree of resistance (maintaining ecosystem function, despite changes) and resilience (the ability to recover to full ecosystem function after disturbance) [81–83].

The south-eastern North Sea is a highly anthropogenic influenced marine area. Since the beginning of the 19^th^ century, the seafloor is continuously affected due to dredging and dumping activities or bottom trawl fishery [84, 85]. Thus, benthic communities of the whole study area are exposed to a continuous disturbance, which might have led to an adapted community structure, which has the ability of a fast recovery and thus stable diversity patterns [86–88]. Another indicator is the high occurrence of opportunistic species with a high reproduction rate such as *Phoronis* spp., *Spiophanes bombyx*, or *Kurtiella bidentata*.

At the most offshore station DB9, two outliers with a significantly lower taxonomic diversity synchronous with a lower trait redundancy were found in 1998 and 2012, and also at the deepest station OG7 in 1999 and 2013. This might be a delayed effect of increased abundances following the extremely cold winter in 1995/96 [64] and the cold winter in 2010 [89]. After both cold winters, increased abundances of opportunistic species such as *Phoronis* spp. or *Notomastus latericeus* were found. Analysis of Reiss et al. [64] found distinct short-term changes in abundance, biomass, and community structure of benthic communities after the cold winter in 1995/96, which were more pronounced in the nearshore areas. Diversity, however, seemed to be more affected in stable offshore and deeper environments.

### Long-term changes in taxonomic and trait-based benthic community variability

Long-term studies such as the present one provide a valuable opportunity to detect and analyse the variability of marine species in relation with changes in environmental parameters [90]. Results of the present study revealed climatic parameters such as SST and NAOI as most important driver variables of taxonomic and trait-based benthic long-term variability, next to epibenthic abundance as a proxy for feeding pressure, and nitrite as a proxy for phytoplankton PP. These climatic and anthropogenic parameters were found as driver variables on other trophic levels [13, 91] and in other marine areas such as the North Atlantic [92] or the Baltic Sea [93].

In the study area, we found distinct changes in taxonomic and trait-based long-term variability of benthic species. Changes around 2000 and in 2010 were congruent with changes in the hydroclimatic regime. However, for the hydroclimatic shift in 2014 no congruent changes in taxonomic and trait-based structures were found. The regimes and shifts in taxonomic and trait-based long-term variability, found in the study area, corresponded to shifts and changes, which were detected in the whole marine and North Sea ecosystem [49, 94, 95].

A high correlation of nitrite with the 1^st^ or 2^nd^ MAFA axis was found at all stations, however, at the offshore stations a predominantly positive correlation was found, while at the onshore stations a negative correlation was found. Differences in the correlation coefficient and in long-term variability of species between offshore and nearshore stations might be another hint for an extensive nutrient limitation in the study area [62, 96, 97]. For sure, due to the basically different environmental conditions, such as sediment characteristics and water depth, the four different benthic communities at the stations react different on environmental changes [98]. Nevertheless, a gradient in N:P limitation [52, 99] and riverine nutrient intake limits primary production and thus food intake in more offshore areas, which is indicated by the positive correlation. The occurrence of one of the most common species in the south-eastern North Sea, the suspension feeding brittle star *Amphiura filiformis* [2, 100], depends highly on food intake. At the nearshore areas, abundances of *A*. *filiformis* increased after 2010, while at the offshore areas a maximum in abundance was found after 2000/01, followed by stable abundances, which correlates with a peak in total dissolved nitrite [36].

Actual state, at the beginning of the present study, were warm-temperate conditions after the smooth BRS in 1988/89 [101–103]. The smooth BRS was characterised by extensive changes in the whole North Sea ecosystem, such as an increase in warm-temperate benthic species with a mainly southern distribution [57]. The phase of warm-temperate conditions was interrupted by a climate shift in 2000/01, which affected the whole North Sea ecosystem as well [57].

Regarding the axes of the MAFA analysis, the regime between 2000/01 and 2010/11 seemed to be more stable in contrast to ongoing increasing values of the nearshore regions and decreasing values at the offshore stations until 2000/01. This was caused due to opposing trends in abundance and BPc of different species and trait groups, respectively. For example, in the nearshore regions of the study area, we found decreasing abundances and BPc of warm-temperate species such as *Scalibregma inflatum* or *Diastylis* spp. and the trait groups B/SM and S/SM, while abundances of *Owenia fusiformis*, or opportunistic tube-living species such as *Phoronis* spp., increased. Furthermore, BPc of trait groups including species with fixed tubes and free movement increased. At the offshore areas, however decreasing abundances of *Kurtiella bidentata* or *Scoloplos armiger* were found. Altogether, comparing climate with taxonomic and trait-based macrofauna variability between 2001/02 and 2010/11, changes seemed to be incoherent, compared with the regime before and after. This corresponds to results e. g. of Dippner et al. [67], which revealed an unpredictable biological time series after the abrupt BRS.

Around 2010/11 single studies indicated drastic changes in the marine ecosystem [43, 89, 104]. Wernberg et al. [43] found a regime shift in a tropical environment after a marine heat wave in around 2011. In the study area, a single cold winter in 2010 [89] interrupted persistent positive SST and NAOI anomalies and increasing SST since the beginning of the study period, which seemed to be a driver variable for slight changes. Overall, after 2010/11 increasing values of the MAFA, and slight increasing abundances and BPc were found. Except for station OG7, which was highly limited by food availability, which might inhibit visibility of climate driven effects.

Canonical correlation analysis revealed feeding pressure through epibenthic species as a driver variable all over the study area and for both approaches. Epibenthic species feed on macrofauna species in a similar range like demersal fish species [105, 106]. In the present study, at more offshore areas with a higher trait-based diversity, feeding pressure is a more pronounced driver variable, than at onshore areas. At the offshore areas higher abundances of highly mobile predators occur [13, 107], which feed on macrofauna species [105]. Most epibenthic species feed selectively, on basis of the relative availability [105]. At Dogger Bank areas (DB9) a higher availability of different trait groups mostly living fixed at the surface, such as *Magelona* spp. and *Spiophanes bombyx* and of mobile species such as *Bathyporeia* spp. resulted in a higher feeding pressure on different trait groups.

### Taxonomic versus trait-based community structure

Recent BEF-research highlights the role of ecosystem functions and diversity for ecosystem stability and resilience [7–9]. Taxonomic approaches, where all species are handled equally, were complemented by trait-based approaches, grouping species which contribute similarly to ecosystem functioning [17, 18, 108]. Still, there are some limitations when particularly considering BPc and our trait-based approach, which uses sediment reworking and mobility traits (Table 1) to create trait groups [34, 35].

Initially, the BPc is an estimate, deriving from existing data, not a direct measurement [34, 35, 109]. Consequently, it is valuable for a large-scale and long-term comparison of existing and consistent data, but for the comparison with other results, however, the theoretical character must be kept in mind. According to Queirós et al. [110], BPc can be used as a predictor for particle distance transport, but it does not give any information on bioturbation depth, activity, or the biodiffusion coefficient Db. Some studies complained about the missing inclusion of functional effects and interactions [108], because most theoretical approaches do not consider important processes, such as inter- and intraspecific species interactions or individual species reactions on environmental changes, which in turn affect bioturbation activities [110, 111]. Nevertheless, within the scope of the present study, it is a valuable option to analyse long-term changes of trait-based diversity and benthic community variability in relation to environmental parameters, especially because of the direct coherence with taxonomic variability.

Overall, concurrent long-term patterns of taxonomic and trait-based benthic community variability in the south-eastern North Sea were found. Moreover, our results confirmed results of previous studies that found similar large-scale patterns of taxonomic and trait-based benthic community structures of three periods from 1986 to 2015 [36, 51]. Despite the concurrent taxonomic and trait-based patterns, our analysis revealed basic long-term changes, next to distinct environmental drivers, between the four stations and between both approaches. Thus, even the trait-based approach based on existing data, it gave new insights, which can be used for further analysis. When considering the most offshore station of the study area, station DB9, taxonomic long-term changes were driven by a variety of species including amphipods such as *Bathyporeia* spp., Nemerteans, or polychaetes such as *Magelona filiformis*. Considering the trait-based approach, most long-term changes can be clearly attributed to one functional group, biodiffursors with slow free movement through the sediment matrix (B/SM). Contrasting, this trait group includes mostly larger individuals such as *Echinocardium cordatum*.
